# ‘What a man can do, a woman can do better’: gendered alcohol consumption and (de)construction of social identity among young Nigerians

**DOI:** 10.1186/s12889-015-1499-6

**Published:** 2015-02-21

**Authors:** Emeka W Dumbili

**Affiliations:** Department of Social Sciences, Media and Communications, College of Business, Arts and Social Sciences, Brunel University, Kingston Lane, Uxbridge, UB8 3PH London, UK

**Keywords:** Alcohol-related problems, Drinking games, Gendered alcohol use, Nigerian students, Social identity

## Abstract

**Background:**

The misuse of alcohol and other drugs among young people, especially students, is a growing global phenomenon. In traditional Nigerian society, different locally-produced alcoholic beverages served complex roles but were mainly consumed among adult males for pleasure. Though adult females in some communities consumed alcohol, the practice of drinking was culturally controlled. In contemporary Nigeria, available quantitative studies reveal changing patterns of alcohol use amongst youth but fail to unravel the social variables that motivate alcohol use among this group.

**Methods:**

Qualitative data were collected through in-depth interviews with 31 (22 males and 9 females, aged 19–23 years) undergraduate students attending a university located in a metropolitan city in Anambra State, south-eastern Nigeria. Data were collected and analysed to generate themes with the aid of Nvivo 10 software.

**Results:**

There appears to be a resilient socio-cultural belief in which men see alcohol as ‘good for males’ while the females in contrast believe that alcohol does not discriminate according to gender and should be drunk by both males and females. Findings also point to the ways in which male-gendered drinking behaviours, such as heavy or fast drinking are employed by women to develop social capital.

**Conclusions:**

These results do suggest how gendered constructions of alcohol consumption create risks for both men and women, how they negotiate and ameliorate those risks, and how women challenge gender roles through their use of alcohol. Some focus on formulating evidence-based policies and comprehensively evaluated campaigns are needed to disseminate information about the risks and potential consequences of heavy alcohol consumption in order to promote safer alcohol use by young people.

## Background

Alcohol consumption among young people has continued to grow in many parts of the world. Studies of students from Western societies [1-3] have provided evidence to show that their drinking is heavier than their non-student counterparts. One of the reasons for this is because heavy drinking amongst student populations is thought to be intimately tied to activities (or behaviours) related to the construction of gender [4,5]. This risky behaviour is facilitated by spaces such as party environments [6,7] in which drinking games occur [8,9].

In the Nigerian context, there is an increase in media reports about students’ misuse of alcohol and other drugs. Alcohol research in this region is nascent, and empirical studies (especially qualitative) that examine the motivations underlying students’ drinking behaviours have not been conducted. This qualitative study begins to address this gap in the literature. Drawing on interviews with 31 undergraduate students of a Nigerian university, this paper reports preliminary findings of an on-going doctoral study. The study has two interrelated aims: to explore drinking motives of party-going undergraduate students, and to examine the use of alcohol for the (de)construction of social identity. The study focuses on the gendered dimension of alcohol consumption to shed light on how men appropriate patriarchal benefits and how women contest hegemonic masculinity through their use of alcohol. The rest of the paper is divided into five sections, beginning with a review of the literature on gender and alcohol. The study’s methods will be detailed, followed by the findings and their discussion. Theoretical and practical implications of the study’s findings will be posited.

### Understanding hegemonic masculinity

Hegemonic masculinity (henceforth referred to as HM) has been a concept widely employed by gender scholars to describe the subordination of women through specific strategies [10]. Demetriou [11] suggests that there are two common types of hegemony: internal and external. External hegemony occurs in patriarchal societies where men’s dominance over women is institutionalized. In these societies, men’s higher social status positions them to have material benefits or ‘patriarchal dividends’ (p2) [12]. Internal hegemony occurs when a category of men maintain a higher social status than other men, thereby establishing the subordination and marginalization of the lower status men. HM needs only a minority of men to police the majority, excluding those categorized under ‘subordinate masculinity’ and women (p832) [10].

Every society has specific behavioural patterns that are categorized as either masculine or feminine and this is often borne out of consensus [4]. Because such behaviours are not inherently biological, individual members of such societies acquire these behavioural patterns through the socialization process. This suggests that gender is not fixed but fluid because ‘gender behaviour is accomplished in the presence of onlookers’ (p742) [4], who categorise such behaviour as masculine or feminine. Given that the formation of hegemonic masculinities is contextual and because social situations change, HM can be altered [10]. Indeed, research has shown that individuals may construct masculinity differently in particular contexts, thereby suggesting that there are *multiple masculinities* [4,10].

According to Connell and Messerschmidt [10], gender relations are borne out of history. Therefore, there is the possibility that hierarchies of gender can change. Humans construct gender through their day-to-day interactions with others [13] and these contextual interactions may necessitate pluralistic masculinities [10]. Thus, through the resistance of, or challenge to patriarchy by women or men that are subordinated by other men, HM becomes subject to change [10]. It has been argued that HM confers benefits to men over women. An example of one such benefit is the different perceptions afforded to the consumption of alcohol by men versus women, where men’s use is less stigmatized than that of women.

### Doing gender with alcohol

A growing body of literature suggests that there is a relationship between alcohol consumption and gender. In fact, the existence of gender differences in alcohol consumption documented in every part of the world suggests that it may be regarded as ‘one of few universal gender differences’ (p1763) [14]. For example, internationally, research shows that young men consume alcohol more frequently than young women [15,16]. Other studies suggest that in many societies, social drinking symbolises masculinity and abstinence connotes weakness [17]. While men often go out to places such as pubs, bars, and so forth with the intention of drinking and to become intoxicated, this is not the case among most women, especially older ones [17]. Given that alcohol consumption is one of the patriarchal benefits available to men, they have greater access to drinking spaces. As such, men have more opportunities for frequent and heavier alcohol consumption than those that are available to women. Another factor that facilitates alcohol misuse amongst men is the association of masculinity with man’s ability to “hold his drink” (i.e., drink but not appear intoxicated) and wider acceptance of men’s inebriation [14].

Although in most societies it is culturally expected for males to drink, studies suggest that a contributing factor to high rates of alcohol consumption (or heavy consumption) amongst men is the interconnection between drinking and other traits associated with the male sex role, such as aggression and risk-taking [18]. Many of the activities in which men engage predispose them to drinking, such as socializing in bars, partaking in sporting activities, and playing drinking games [19]. Alcohol consumption is thought to increase in these contexts because it forms the basis upon which a man’s masculinity is evaluated by other men [4]. Further, gender does not just determine how people use substances such as alcohol, consumption of alcohol and other drugs can be used to *do gender* [20,21]. That is, alcohol consumption is used to fulfil gender roles and expectations [22].

Among young people, studies have revealed a variety of motives underlying drinking behaviours, such as self-enhancement, [23] and the expression of masculinity [24,25] and femininity [26]. Similarly, studies conducted among student populations revealed that female [5,27] and male students [4,28] use alcohol heavily and scholars argued that alcohol consumption is one way students express masculinity on campus [4,28,29]. Although alcohol consumption is one of the ways people do gender, how, when and where this is done differs amongst different groups. For example, while some individuals may use heavy consumption to distinguish their drinking (and themselves) from other groups [4], others may employ measures to regulate their drinking in order to not exceed limits that act as markers of gender identity [21].

Despite that the enactment of hegemonic masculinities may confer presumed advantage to the victor (men), it does not come without a cost [30]. This is because physical and emotional damage can result [10]. In terms of alcohol consumption, a number of studies have shown that although doing gender with heavy episodic drinking may give ‘liquid courage’ (p747) [4] to the drinker, it often results in physical, emotional, health and social problems [28,31-33]. Despite that the use of heavy alcohol consumption to construct gender identity has dire consequences, it is surprising that in some countries, females are said to be constructing gender identity with heavy alcohol use in contemporary society.

### Feminization of heavy alcohol use

The literature suggests that women who drink in places where their drinking is socially taboo are seen as unfeminine [18], feckless [34] or as guilty of having transgressed femininity [35]. A reason behind the tensions surrounding women’s drinking or excessive use of alcohol is the belief that such behaviour does not affect her alone. Instead, a woman’s problems with alcohol are thought to affect her children, the family, and society at large, and therefore, her use of alcohol prompts an outcry [14]. Different studies [36-38] have provided biological explanations on the effects of alcohol on the human body, stating that females are more vulnerable [39], but this appears to be over exaggerated or ‘magnified by cultural norms for how women and men should or should not use alcohol’ (p742) [4].

Although evidence shows that men continue to dominate drinking spaces [40] and consume greater quantities of alcohol [41,42], research suggested that females now binge and engage in other forms of heavy drinking rituals [5]. The erosion of traditional value systems that hitherto hindered women from drinking [43] and the ‘feminisation of night-time economy’ (p188) [26] are thought to be reasons underlying these changes. Other reasons include the quest for self-expression [44] and the increase in females’ employment, which results in women spending less time at home [14]. Other researchers have argued that females are not just venturing into areas previously dominated by males (such as drinking games and other public drinking spaces), they are now drinking what was previously tagged as being ‘males’ alcohol’ such as spirits [45].

In countries where women’s alcohol consumption is growing (such as Finland), [46] studies have shown gender convergence in alcohol consumption patterns. According to Bloomfield et al. [46], factors responsible for this include the increase in females’ socioeconomic status and the increased advocacy for female emancipation. Women are challenging patriarchal beliefs about gendered alcohol consumption [26] such as those inherent in drinking discourses that are centred on ‘risk, responsibility and appropriate behaviours when drinking’ (p4) [35], particularly within public spaces. It has also been reported that while young men engage in substance use as a way of doing gender, substance use among their female counterparts is a source of gaining cultural capital [47].

These remarkable changes in consumption patterns amongst women have prompted increased scholarly attention, particularly in Western countries. In the USA for example, Lemle and Mishkind [18] view the upsurge of female emancipation in the 1970s as responsible for this shift because it brought about a relaxation of the stringent ways of defining male and female sex behaviours. In Australia, Palmer [35] suggested that drinking practices are some of the legitimate means through which females express their identities as sports followers and supporters. It is also argued that females may not drink less or abstain because they have been one of the major targets of alcohol producers [14]. Because researchers have neglected the fact that women in many countries are now drinking in spaces considered to have been hitherto occupied by men (such as in bars, pubs and sports venues), their drinking problems appear to have continued to be underestimated. This underestimation may hinder proffering of holistic solutions. Thus, Palmer (p9) [35] advised that the concept of HM (where it is assumed that only men drink heavily) should be rethought. She argued instead, that ‘hegemonic drinking’ (i.e., the use of heavy drinking amongst men and women to construct gender identity) should replace the former.

### Alcohol and the Nigerian society

It is thought that the perception of the role of alcohol in a society is related to that society’s specific inherent normative structure. How each society perceives alcohol determines what it is used for, who drinks it, and how it should be consumed [48]. While some societies may see alcohol as ‘part of sociability’, others may see it as ‘a special product to be controlled’ or even as a ‘moral threat’ (p1766) [14]. Nigeria is a patriarchal society in which women most commonly experience subordination and marginalization. One of the ways patriarchal relations manifest is in the male presumption of their right to consume alcohol. Alcohol consumption may be seen as a means available to men to display their discrimination against women and negate women’s position in the social relations.

In traditional Nigerian society, locally-produced alcoholic beverages such as *palm wine* (sap from palm trees or raffia palm), *burukutu* and *pito* (fermented from Sorghum or maize) served complex roles in social rituals such as the opening and closing of family meetings, ancestral worship, and the consummation of marriage, amongst others. Adult males were expected to drink at such occasions and their failure to do so would be responded to with suspicion [49]. However, outright hedonistic consumption was considered taboo in many communities [50] and alcohol consumption among young people was culturally restrained [51]. The weight of taboo against women’s alcohol use has been so significant that marginalization and stigmatization of a woman drinker extends beyond herself to include her family. For example, if a woman is thought to consume heavily or inappropriately, her sisters’ chances of attracting a suitor will be greatly hampered [52].

In contemporary Nigeria, available quantitative studies reveal that many young men are drinking hazardously and are using other substances [53,54]. Among their female counterparts, a few studies [55,56] have identified different patterns of alcohol use. Obot [57] attributes the rise in women’s consumption to pervasive alcohol marketing that targets women with female-friendly beverages. However, this does not address motivations for drinking among women– an issue unexplored until this current study. Again, while a plethora of studies on alcohol and gender construction among students in Western countries exists, a scan of the literature suggests that none have been conducted in Nigeria. More specifically, no study was identified that employed qualitative methodology to explore gender construction, alcohol use and perceptions of use amongst student populations in Nigeria. These gaps suggest that there is a pressing need to empirically examine what motivates young people in Nigeria to drink alcohol and to use alcohol to (de)construct gender identity.

## Methods

### Procedure and ethical approval

The study was conducted in a university campus located in a metropolitan city of Anambra State, south-eastern Nigeria. Prior to data collection, ethical approval was awarded by the Office of the Dean of Students’ Affairs of the Nigerian university and the Brunel University London Ethics Board. The Office of the Dean of Students’ Affairs of the university in Nigeria was responsible for reviewing and approving studies concerning students at the time the ethical approval was awarded in 2012. Data were collected between September and December 2013. The participants were recruited across the nine faculties in the university campus using word-of-month and snowballing techniques. It should be noted that these recruitment processes were adopted as alcohol consumption among young people is a sensitive topic in Nigeria and as such, young people, especially females, are not easily recruited for such studies because of their fear of identification. In order to protect their confidentiality, all names used here are not the participants’ real names. Additionally, in accordance with BioMed Central editorial policies for reporting qualitative studies, the author confirms that this article has adhered to the RATS guidelines.

### Participants and interviews

Thirty-one open-ended interviews lasting approximately 33–90 minutes were conducted with 22 male and 9 female undergraduate students, aged 19–23 years. Although eliciting more data from females would have provided greater nuance, some recruitment difficulties hindered the inclusion of more women. A significant barrier to the recruitment of more women was the greater stigmatization of female drinkers, even though alcohol consumption by young males and females is socio-culturally disapproved in Nigeria. As such, some potential female participants that were approached during the fieldwork declined the invitation to participate. The interviews were recorded with a digital device with the permission of participants. All but one of the participants is from Igbo tribe (an ethnic group that lives in the region where the study was conducted). Participants were invited to select the location of the interview. While four of the interviews were conducted on the school campus, 27 (87%) were conducted in participants’ off-campus hostels. In order to ensure voluntary participation, no incentive was given to the participants.

After testing the interview protocol in a pilot study, an interview schedule was developed. It consisted of 12 main questions that were expanded through probes during the interviews. Each interview began with an effort to establish rapport and obtaining demographic information. The questions that followed sought to elicit from them their perceptions and beliefs about alcohol and the meaning it holds for them. Participants were also asked about the quantity of alcohol consumed, the frequency and locations of consumption, and with whom they most frequently drink alcohol. Additional questions explored the participants’ perspectives on students who abstain or drink little alcohol, and what they consider to be “normal” consumption for males and for females at a drinking occasion. Determining amounts consumed was challenging given that Nigeria does not have alcohol policies in which a standardized drink is defined [58]. Instead, participants were asked about the number of bottles or glasses of alcohol they consumed.

### Data analysis

The interviews were transcribed verbatim and a thematic analysis was undertaken [59]. As recommended by Silverman [60], preliminary analysis was initiated immediately after the first interview was conducted in order to guarantee quality and timely analysis. Field notes and audio recordings were reviewed to check for accuracy and to identify additional areas to explore further in subsequent interviews. Tentative coding schemes were developed at this early stage [59]. Following this, the first interview was transcribed and anonymised, and initial extracts were categorized into broad themes and subthemes. This process was repeated for the next six interviews. A collaborative approach to analysis was undertaken in which the study’s team of academic supervisors reviewed and commented on selected interviews and the initial thoughts and ideas about coding and themes. This turned out to be very useful, in that it assisted me to have an early grasp of my data [61] and some of these subthemes grouped manually became the parent nodes while others were condensed [62] into different child nodes that formed the thematic coding framework when the data were imported into Nvivo 10. When the 31 interviews were completed and transcribed, the author completed the thematic analysis.

## Results

The results presented here centred on the motivations underlying men and women’s consumption of alcohol and the use of alcohol to (de)construct gender identities. Related to these findings are the use of particular brands of alcohol to do gender, and men’s reactions towards women’s use of alcohol. Key themes informing these findings pertain to the ways in which women contest stereotypes associated with femininity and drinking practices. Specifically, five themes are considered: (1) women’s motivations: drinking for pleasure and sociability; (2) men’s motivations: courage and machismo; (3) alcohol as markers of masculine identity; (4) alcohol does not discriminate according to gender, and (5) gendering of alcoholic beverages.

It is noteworthy that all the participants were current users of alcohol but their drinking patterns, frequency of consumption and motivations for drinking varied, often in ways that related to the meaning of alcohol to each participant. Among the participants, “drinking stories” were constructed around *who* should drink, *what* kinds of alcohol should be consumed and *why*. Males’ drinking stories related alcohol use with the acquisition of social capital. They described two sources of social capital related to drinking: most popular was the importance of consuming a large quantity of alcohol without showing signs of intoxication, and the second was becoming the “fastest drinker”. Motivations for drinking described by females were largely social.

### Women’s motivations: drinking for pleasure and sociability

Among the women who use alcohol for social reasons, alcohol is mainly consumed only occasionally, such as parties and in small quantities. Not only is alcohol use in such occasions considered to be ‘normal’ [6], female social drinkers suggest that drinking alcohol gives pleasure and make these occasions:

When you go to parties, you don’t expect them to give you juice or soft drink. What you need is alcohol and when you take alcohol, it will make you feel the groove, it will make you be in the spirit of the party so you can dance…you just end up being happy. (Genny, female)

Following on this, another female participant confirms that alcohol consumption at parties is primarily for pleasure, to brighten people’s moods, and to enhance their sociability:

If people in a party are consuming only juice, everywhere will be dull and some people will not misbehave; so it will not be fun. You know that some people are shy but when they take alcohol they tend to dance…(Chioma, female)

### Men’s motivations: courage and machismo

All the male participants (n = 22) agreed that they drink for social reasons, but they equally have other reasons for using alcohol in parties. While the majority corroborated what the female participants said about the importance of alcohol being present at a party, their drinking motivations in a party environment varied significantly and differed from that of females. Here, alcohol does not just facilitate the opportunity to let down their guard; rather, alcohol seems to provide them with courage to engage in sexual negotiations:

Alcohol gives you the boldness and enhances the spirit. You might see a lady and maybe you might have been eyeing her for a long time and you don’t have that gut to walk up to her… But with a bottle of beer or two, you will feel as if the world is under your feet. It drives away fear…you will be able to walk up to the girl and tell her what you want. (Okezie, male)

Additionally, over 80 percent of the male participants stressed that alcohol in parties does not only facilitate sexual negotiations, but it also provides an opportunity to show off masculinity and gain social capital. This is because men believe that alcohol is supposed to be used by only males because they are strong, while women, whom they consider to be fragile, should not drink. Contrary to Peralta’s [63] assertion that men’s reaction toward women’s heavy drinking would not be the same had women engaged in light or moderate drinking, the majority of the men in the context of this current study did not consider women fit to use alcohol in the first instance. The perception that alcohol is inappropriate for women to consume is further supported by the absence of women’s participation in drinking games, a ritualistic sport used by men to demonstrate their masculinity. In this male-dominated context, drinking games are played largely by heavy and fast drinkers who are almost always men. Popular games involve contests to determine the fastest drinker, or who is able to drink the most without vomiting or giving up:

In parties, you will see guys who will come out and show themselves (as real men)…Actually I have witnessed stuffs like that where guys would normally volunteer themselves to drink beer (provided by the host)…the highest drinker or the fastest actually wins it…It’s just getting that bragging rights over others that ‘I drank and you couldn’t’ that makes boys do it…(Boniface, male)

Additionally, Boniface’s words shed light on the way in which men are admired by both male and female party attendees when they perform this male gender appropriate behaviour (i.e., by publicly displaying their superior masculinity in game playing). The resultant fame or popularity achieved facilitates flirtation and sexual encounters, and as such, appears to be a strong motivation for participating in drinking games.

Another participant, Buchi, makes a statement that supports the idea that boys use game-playing to demonstrate their superior masculinity and gain more social capital:

What they get is that they make people know that when it comes to drinking, you’re the boss. People grade you by the level of drink you can take. I mean the number of bottles you can consume. So people know that when it comes to drinking, ‘this guy is the boss’…(Buchi, male)

Further evidence that demonstrates that drinking games in mixed-sex parties are exclusively for males was provided by the females. In fact, all female participants stressed that they have never participated and indicated that they perceive such participation to not be normative for females. Although three (33%) out of the nine female participants argued that they can even drink more than many males, drinking beer or competing in such parties would be seen as unfeminine and a ‘failure to do gender appropriately’ p.375 [64]:

The society sees Star, Gulder, Legend (popular beers in Nigeria)…as masculine. So seeing a female take it, even I myself sees that person as indecent. (Pretty, female)

These findings shed light on some of the reasons for participating in (or for women, reasons for refraining from participating in) drinking games. Although the results of drinking games provide social capital for sexual negotiation and manipulation, they also contribute to the popularity of hazardous drinking among students. In turn, this supports the claim that practices of HM do not always result in what is in one’s best interest in terms of one’s health. Thus, it suggests that health is a secondary concern to HM, in that the very notions of HM impede men’s interests in caring for their health, (i.e., masculinity and health-promoting behaviours are in tension) [11].

### Strong men and fragile women: alcohol as markers of masculine identity

A second finding of interest was the way in which students expressed their beliefs in who should or should not drink, and what type of drink is appropriate for whom. Just as a study conducted among students in the USA [63] reveal that race and gender influenced perception as to who should use alcohol (and how it should be used), men in this current study drew on their socio-cultural beliefs (based on Igbo culture) about gender relations to support their reasons for who they felt it was appropriate or acceptable to drink alcohol. In answer to the question about attitudes towards males’ and female friends’ use of alcohol, the majority (68%) of men stated that alcohol should be consumed by men only. Examples of phrases employed include: “alcohol is for the guys”, “it is men’s world”, “Nwoke-adi-njo” (translation: “nothing a man does is really bad”), and “it is not in our culture for a woman to get drunk”. When asked about why he thought only men should drink, an undergraduate law student argued:

It has to do with culture, Igbos generally have the belief that women are restricted, so they don’t take alcohol…People normally have the perception that only men should take alcohol. Women are restricted from taking alcohol because once a woman starts taking it, the society will start having the impression that ‘look you are getting out of your bound’. (Kelly, male)

From this excerpt, it is clear that this student drew upon socio-cultural beliefs about the role of women and their place in the Nigerian society to explain his reasoning behind his ideas about women’s consumption of alcohol. In general, representatives of the formal social structure in contemporary Nigeria (such as the police) would not respond negatively to women’s consumption of alcohol, provided that they did not violate any laws related to alcohol such as the legal drinking age. However, the informal responses to their drinking strongly discourage women from using alcohol [63]. These different reactions to women’s use of alcohol work to position women who do drink as those who engage in ‘non-traditional gender practices’ p.377 [64]. As such, these contradictory reactions can exacerbate social inequality by expanding gender hierarchies, which further disadvantage women.

While some want women not to drink due to prominent socio-cultural beliefs about women’s place in the society, a substantial number of men argued that women’s inherent characteristics (e.g., women are weaker and more fragile than men, they are at risk of physical and moral harm) preclude them from drinking. Here, they stressed that alcohol is meant for the ‘strong’, making it inappropriate for women whom they characterized as ‘fragile’ or ‘people with light brain’ to drink:

I know that if males take alcohol, it is good because they can control themselves because they are guys. For females, if they take alcohol, they might end up getting drunk and might easily derail. (Diogor, male)

Similarly, some men justified this notion by drawing on ideas of ‘respectable femininity’ [65] to argue that it is unfeminine for a woman to get drunk. For women who do drink, there is an imperative that they avoid intoxication, or at minimum, avoid ‘obvious intoxication’. While consuming alcohol is generally not considered appropriate for women, intoxication is regarded as a form of ‘female-specific deviance’ (p394) [63]. This is further illustrated in the following statement:

First of all, it is kind of pathetic to see a woman that is drunk…For the male to be drunk, it is not too heard off (not always publicised)…but for a woman to be drunk, it is not morally good…(Larry, male)

Not only do men view alcohol as good for men only, they also view alcohol consumption as a way of determining amongst themselves “who is a *real* man”. For example, the amount of alcohol a man can consumed will determine his rank amongst his male peers. On the other hand, those who abstain from drinking are not considered to be “real men” [18,24]. An interesting part of this display of masculinity is the emphasis on the quantity of alcohol consumed as measured by the number of bottles. Thus, a male participant described his tactic of changing his brand of drink from a stout that had 7.5% alcohol by volume (ABV) to one with 5% ABV. This heightened his status amongst his peers by allowing him to consume larger quantities of alcohol. Because among his friends, drinking the same brand helps in sharing camaraderie [42], while the number of bottles one consumes is used to display badges of honour:

There is this masculine ego that comes into play even before we head to the bar. Like, how many bottles do you think you can finish? Do you think you can do four or five? Hey I’ve drunk seven bottles…So you don’t expect after making such brags, you go there and you just take only two bottles…Instead of…drinking only two bottles and feeling less than a man in the presence of your colleagues, which means that they will make jest of you for a while, it’s better you go for Star beer that has less alcohol content, so that I can drink more bottles and feel manlier…(Edulim, male)

In the excerpts above, it can be inferred that male participants believe that alcohol should be consumed by men. Further, they justify their beliefs using moral arguments and advance the idea of ‘being strong’ to support their acquisition of patriarchal dividend in the form of alcohol consumption.

### Alcohol does not discriminate according to gender

As mentioned above, six of the females participants believe that it is acceptable for men to drink alcohol but not for women. Further, it appears that their beliefs are mediated by cultural notions that women who drink are ‘wild girls’ and ‘unfeminine’. Three other female participants expressed beliefs that suggest that alcohol does not discriminate according to gender, and so it does not matter if it is a woman or a man consuming it. To them, it only matters if someone is ‘strong enough’ to take it. For example, Patience emphasized that the issue is not gender, but strength or capacity to consume that is relevant to the appropriate consumption of alcohol (i.e., appropriate consumption is limited to a level at which one does not become inebriated).

In addition, these female participants expressed several reasons as to why women should be able to drink. The reasons given include gender equality and pleasure. They argued that gender equality implies that women have (or should have) the right to drink what they want, a right that is bestowed socially rather than ‘by alcohol’. For example, one of them argues that she drinks to display her badges of honour [4] among peers by showing them that she can hold much alcohol:

I can take more than three bottles and still feel okay…I’ve always known myself to be a strong person and when I’m determined to do something I don’t look back…So I don’t let myself get down before my friends. I don’t like them seeing my weak point; so I try in order not to get tipsy in their presence. (Pretty, female)

Indeed, Pretty sheds light on how she developed a capacity to “hold her drink”, by revealing that she has always challenged men in diverse activities even before she gained admission into the university. Interestingly, although she uses heavy drinking to do gender and to challenge gender norms and expand categories of what is acceptable behaviour for women, she also strives to maintain her personal respect and integrity among peers in order to avoid being categorised among ‘bad women’ (p396) [63]. To accomplish this precarious position, she must strategically manager her consumption so that she does not become intoxicated, or at least not display signs of intoxication.

In fact, from her account, it appears that this ability to hold her drink increases her rank among her peers, whom she described as those who often show signs of intoxication after consuming fewer bottles than she does.

Additionally, another female participant stressed that the quantity of alcohol consumption between males and females should be comparable because men and women are both human beings. As such, what men can handle, women should too:

I believe the quantity should be the same. Why can’t ladies drink alcohol the way guys are taking it? Because funny enough, you see some guys that cannot even take more than two bottles but some ladies can take 10. There are some ladies that when it comes to drinking, you cannot even defeat them…(Chichi, female)

Similarly, when asked what she thinks about cultural beliefs that restrict women from drinking alcohol, Chichi noted that she was aware of these beliefs and argued that such beliefs create the circumstances that force women to engage in secret or hidden drinking. Thus, she argues that men should not restrict alcohol consumption to themselves because not only are women moving into similar rates and types of employment as men, some women are achieving the same or higher status than some men [46]. The excerpts below show Chichi’s feminist view as well as that of another female participant, Ada:

Why can’t a woman do what a man is doing? …Come to this contemporary world, I don’t believe there is anything a man can do that a woman cannot do. Men are ministers and women are ministers;…in some countries women are president. Men and women now work in the same company, doing the same kind of work…In some houses, women are making more money than the guys… So why should you come and tell me that a man should drink alcohol and the woman should not drink even if she likes to drink alcohol?…(Chichi, female)

When I see (Nigerian) girls going for that…(alcohol and other risky events), they are like telling the people in the world that what a man can do, a woman can do better. (Ada, female)

As HM is often contested as children grow up, individuals have the ‘capacity to deconstruct gender binaries and criticise HM’ (p853) [10]. As these excerpts reveal, this can be said to be what young people, especially the females, are doing with alcohol consumption in contemporary Nigeria. Despite that, unlike what was found among American female students who use heavy drinking as an opportunity to violate the boundaries of traditional gender norms and excuse their behaviour by blaming alcohol [64], Chichi and other women who use heavy drinking to do gender do not want to lose control as a result of alcohol; thus they continued to discourage being “out of control”.

### Gendering of alcoholic beverages

The previous section addresses the ways in which alcohol use is gendered and how its use has different implications and opportunities for the construction of gender identities. This section drives this point further by demonstrating how alcohol has itself been gendered (that is the substance, not just its use). The majority of the male participants differentiated between “males’ or guys’ alcohol” (such as beer, spirit, gin) and beverages that are more appropriate for females. Again, cultural beliefs about males’ and females’ roles were employed to support their reasoning behind their categorization of alcoholic beverages as either appropriate (or not) for men and women:

In this eastern region, and in (name of city) to be precise, when you see a girl drinking Gulder (beer)…, people will say ah, what is this girl doing? It’s abnormal, it does not make sense, it’s not good; she supposed to be taking something like malt (non-alcoholic) and leave Gulder for men to take…So they see it as totally abnormal that a young girl should be taking beer (Ejike, male)

A different picture was painted by a few males who believed that it is acceptable for women to drink. However, although they felt it was okay for women to consume alcohol, they stressed that there are only certain types of alcoholic beverages that are appropriate for women. In general, beer was not considered to be appropriate for women, except for when taken medicinally (e.g., stout that is thought to ease menstrual pain in Nigeria). Beer was described as causing men’s stomachs to protrude, a side effect that would be unbecoming for women, thereby making beer inappropriate for women to drink. Although spirits were not considered to be a woman’s drink, one of the male participants argued that they were preferable to beer because spirits would help women maintain their “twiggy” figure. By and large, the alcoholic beverages deemed appropriate for women were those perceived as sweetened or “light” such as wine or Smirnoff Ice. In fact, the men added that such beverages shouldn’t even be considered as alcohol due to their sweet tastes and presumed very low ABV.

Although women expressed a greater diversity of opinions than men, many did support the assertion that bitter alcoholic beverages or those with a high ABV should be left for the men. For example, Chisalum believed males should drink beer:

Chisalum: …It is bitter and it is for guys. In this campus, I have never seen a female drinking beer. Rather, they drink red labels like Night Train (flavoured wine with 17.5% ABV)…but to open bottles of beer…girls don’t drink it. They drink wines and drinks like Smirnoff, not beer.

Interviewer: Why do you think girls go for wine and Smirnoff while boys drink beer?

Chisalum: …Like I said earlier, the culture (determines what to drink); the trend is that males drink beer. If you are a female and you are drinking beer, people feel you are irresponsible and you lack home training…

All the female participants primarily consumed sweetened alcoholic beverages (due to the taste and or the fear of people’s reactions if they were to drink beer). However, one participant said that in addition to drinking sweetened beverages, she also consumes spirits. For example, Chimanda stated a preference for spirits because she felt that spirits make her ‘**‘get high quicker”** and were better for maintaining her figure because they do not cause her stomach to bloat. Similarly, Pretty (a self-described “female alcohol macho”) drinks Smirnoff Ice, but of interest is that, unlike most other participants, she is aware of the risks related to consuming sweetened alcoholic drinks. For example, she argues that although society is most accepting of women drinking sweetened alcohol, most of these types of beverages contain more ABV than many beers that men drink:

…Like Smirnoff ice which is the…most popular alcohol for ladies…is about 5.5%…The taste is a little bit mild but the alcohol percentage is equivalent to or higher compared with most of the beers that taste bitter…The truth is that most people don’t know the alcohol percentage in Smirnoff Ice… So when adults see you taking it, they think you are just taking a soft drink because of the appearance, but we know it has high alcohol percentage…(Pretty, female)

She added that she does not support the dominant view that females should only drink sweetened alcohol. Instead, she expressed her opinion that women should be allowed to drink whatever they want, whether it is bitter or sweetened.

As these accounts suggest, some women consume beverages that are gendered as women's drinks, however some of them (and other men) are unaware that these drinks tend to have a high ABV. The extracts also show that females are beginning to contest these patriarchal drinking practices, and there is a growing consciousness that irrespective of one’s gender, individuals should be allowed to choose the type of alcoholic beverages they want to drink. Again, as it has been exemplified above, alcohol consumption may appear to be lower among women, especially in societies where their drinking is restrained or totally stigmatized [66], but such restriction may open doors for different types of alcohol-related problems. In the Nigerian case, females may be drinking more potent alcohol with high sugar content than men presume and this they have no control over because the society sees sweetened alcoholic drinks as a woman’s beverage.

## Discussion

The results from this study shed light on the gendering of alcohol consumption among a group of 19-23-year-old university students in Nigeria and contribute to the existing literature on the use of alcohol in the construction of social identity. The findings support Frederiksen et al.’s [6] assertion that alcohol consumption is associated with pleasure and celebrations by demonstrating that female students are mainly social drinkers who derive pleasure in moderate drinking in parties. While women’s drinking is pleasurable, men view parties as opportunities to acquire social capital and present themselves as “drinking machos” by participating in drinking games. Part of their success in accomplishing this drinking macho status is achieved by restricting women from such game playing, thereby allowing the patriarchal dividend to be accrued to themselves. In doing so, they do not just impress other men, they also impress women and open up opportunities for sexual negotiations with women.

This supports Johnson’s [67] and Pedersen’s [68] findings that sexual reasons often engender game playing. It has been revealed that although hegemony does not connote violence [10], it may be bolstered by force and possibly be achieved via persuasion. By using cultural gender norms to restrict the females from contesting and drinking alcohol, men perpetuate male dominance in alcohol consumption in contemporary Nigeria. In fact, there are social and health implications of this for women: alcohol related risks are enhanced by their use of sweetened beverages due to their high ABV, and others are pushed to drink in secret. Indeed, this extends Peralta’s [63] findings that students from a racial group altered their public drinking behaviour to prevent being labelled negatively by demonstrating that a similar phenomenon occurs for women given their lower status within the same ethnic group.

Similarly, as the findings reveal, males do not just restrict alcohol as appropriate for consumption for themselves, they also use heavy drinking (measured in the number of bottles consumed rather than type of alcohol consumed) to display their masculinity and to distance themselves from inferior masculinity or “lesser” men. In this, we see what Demetriou [11] called ‘internal hegemony’ being played out, in that some men distinguish themselves from other men by the number of bottles they can drink. To prove that one is a “real man” among his peers, he has to participate in binge drinking by taking as many bottles as possible and if one does not drink at all or drink fewer bottles, he is said to be “dulling” (deviating from the group norm as well as reducing the fun of the group) and thus is ridiculed by other men. Although Mullen et al. [25] reported that participants in their study were aware of the consequences of becoming intoxicated, this current study paints a different picture because social risk (i.e., being ridiculed) outweighs health risk. The struggle to maintain a masculine identity or to acquire more social capital superseded concerns about alcohol-related risks. This reinforces the suggestion that drinking motives often blur the perception of the consequences of risky drinking behaviour amongst youth [69]. An interesting strategy used by men to maximize the amount of bottles consumed is the substitution of a beverage with a high ABV with a brand that has a lower content. This enables the man to remain competitive with the number of drinks consumed (whether or not this reduces alcohol-related harms is a question for further empirical study).

This study also explores women’s diverse drinking patterns and the motives behind them. The study’s findings on the themes of women’s use of alcohol and gender constructions confirm and extend the findings of other studies conducted outside of Nigeria. Lemle and Mishkind [18] argued that HM requires men’s avoidance of sweetened alcohol, a beverage suitable only for women. In this study, we see that women share this view. However, females drink these sweetened alcoholic beverages not only because HM dictates that they are appropriate drinks for them, but also because they prefer the taste. An important consideration leading women to choose to consume ‘women’s drinks’ rather than a ‘man’s drink’ is the fear that they will be labelled “wild girls” or considered unfeminine if they drink beer.

An unexpected finding of this study was that none of the men interviewed were aware that many of the ‘women’s drinks’ (such as Smirnoff Ice and wine) contain greater amounts of alcohol than ‘men’s drinks’. For example, Star (the most popular beer among men) contains 5% ABV (in 600 ml bottle) but Smirnoff Ice and Orijin (the most popular pre-mixed beverages among women) have 5.5% and 6% ABV (in 300 ml bottles) and some wines (e.g., Night Train) have up to 17.5% ABV. While the average male in this study mentioned that he drinks five bottles, some females took between three to five bottles of Smirnoff Ice while two females drink between one and three bottles of wine in a drinking occasion. Thus, gendered notions of alcohol as it relates to femininity produces risks and harms for women, and that the social relations that reinforce the gendered constructions of alcohol and its use make these risks difficult to identify [70], in that women’s alcohol use will be underestimated. Relatedly, they also make women exposed to high sugar intake because of these sweetened drinks.

The current study has also shown that some women are beginning to question the assumption of masculinity associated with the amount of alcohol consumed. This reinforces Palmer’s [35] assertion that *hegemonic drinking* should replace HM. According to Holmila and Raitasalo (p1767) [14], women’s entry into the paid workforce may contribute to their use of alcohol in that along with employment, they adopt other ‘male values and behaviour patterns’ and experience an increase in ‘freedom as individual consumers’. Similarly, women have been recognized as a new consumer market by alcohol industries and the development of sweetened alcoholic beverages targets women specifically [57]. As Connell and Messerschmidt [10] note, not only does HM change, the geography of masculinities can also change. In the Nigerian context, since its independence in 1960, developments of the state have contributed to changes in gender relations. For example, women are increasingly visible in managerial positions in organizations thereby requiring men to respect their ‘managerial masculinity’ (p853) [10]. Such transformations in gender relations may be partly responsible for the changes in alcohol consumption amongst women.

## Conclusion

This study shows how gendered constructions of alcohol consumption create risks for both men and women (i.e., both health risks, such as over or hidden consumption, and social risks, such as being seen as not properly occupying their assigned gender roles). It also shows how men and women negotiate and reduce those risks (e.g., men change to drinks with a lower alcohol content; women drink in secret and drink sweetened beverages to ameliorate social risks at the expense of potentially increasing health risks), and how women challenge gender roles through their use of alcohol.

The study is limited by the fact that fewer females were interviewed and thus, their views may not represent those of other young females. However, in keeping with qualitative methodology, a representational sample was not sought, and the findings are not meant to be generalizable. Additionally, the data were collected from a university campus and as such, the views expressed here may not represent that of young people from the region or from Nigeria more broadly. However, this study makes important contributions to gender and alcohol research by studying an understudied group using qualitative research methods, which are underutilised among alcohol scholars in Nigeria [71]. The study also provides direction for further research. For example, because studies outside Nigeria have revealed that people’s drinking habits change as they grow older, qualitative studies that involve older adults should be conducted in Nigeria to further explore these themes.

Theoretically, the findings support the argument that the concept of hegemonic drinking is more fitting than the concept of HM [41] because it offers greater nuance to the more general concept of HM. This theoretical argument has important practical implications, in that until it is recognised that women in contemporary Nigeria consume alcohol, their use will remain hidden and they will be at increased risk of alcohol-related problems. Acknowledging hegemonic drinking opens the door to the development and implementation of effective solutions to address both health and social risks.

Other practical implications arise from the findings that demonstrate widespread ignorance, misunderstanding, and misinformation about the alcohol content of beverages and the related risks associated with alcohol consumption. Such lack of knowledge and confusion is likely related to the absence of policies to regulate the size of standard drinks, the potency of alcoholic beverages, and their marketing in Nigeria [72]. As such, this study supports the formulation of evidence-based policies in these regards. Further, the findings suggest a need for the development of guidelines to inform drinkers about the risks associated with the consumption of sweetened beverages. Finally, widespread comprehensively evaluated campaigns are needed to disseminate information about the risks and potential consequences of heavy alcohol consumption in order to promote safer alcohol use by young people.
